# Physical capacity modulates intestinal barrier dysfunction in functional disorders: phenotype-specific patterns in fibromyalgia and irritable bowel syndrome

**DOI:** 10.3389/fphys.2025.1695163

**Published:** 2025-10-29

**Authors:** Francesco Russo, Antonella Bianco, Laura Prospero, Gaetana Laselva, Marco Grasso, Benedetta D’Attoma, Nicola Verrelli, Antonia Ignazzi, Isabella Franco, Francesco Goscilo, Claudia Beatrice Bagnato, Anna Ancona, Maria Notarnicola, Michele Linsalata, Giuseppe Riezzo

**Affiliations:** ^1^ Functional Gastrointestinal Disorders Research Group, National Institute of Gastroenterology IRCCS “Saverio de Bellis”, Castellana Grotte, Italy; ^2^ Laboratory of Movement and Wellness, National Institute of Gastroenterology IRCCS “Saverio de Bellis”, Castellana Grotte, Italy; ^3^ Rheumatology Outpatient Clinic, National Institute of Gastroenterology IRCCS “Saverio de Bellis”, Castellana Grotte, Italy; ^4^ Outpatient Pain Management Unit, National Institute of Gastroenterology IRCCS “Saverio de Bellis”, Castellana Grotte, Italy; ^5^ Core Facility Biobank, National Institute of Gastroenterology IRCCS “Saverio de Bellis”, Castellana Grotte, Italy; ^6^ Laboratory of Clinical Pathology, National Institute of Gastroenterology IRCCS “Saverio de Bellis”, Castellana Grotte, Italy

**Keywords:** fibromyalgia, irritable bowel syndrome, physical capacity, gut barrier, dysbiosis

## Abstract

**Background:**

Intestinal barrier dysfunction is increasingly implicated in the pathophysiology of fibromyalgia (FM) and irritable bowel syndrome (IBS), particularly in their comorbid form. Physical capacity (PC) influences systemic inflammation and metabolic resilience, but its relationship with gut barrier integrity in these conditions remains poorly defined.

**Methods:**

We conducted a cross-sectional study of 56 patients with FM (n = 14), IBS (n = 23), or both (FM + IBS, n = 19). Intestinal barrier function was assessed using serum and fecal zonulin, intestinal fatty acid-binding protein (I-FABP), and the lactulose/mannitol (Lac/Man) urinary excretion test. PC was quantified using the Global Physical Capacity Score (GPCS). Clinical symptoms were evaluated using the Fibromyalgia Impact Questionnaire–Revised (FIQ-R) and IBS Severity Scoring System (IBS-SSS).

**Results:**

FM + IBS patients exhibited the greatest barrier dysfunction, with elevated fecal zonulin and Lac/Man ratio. IBS patients showed increased I-FABP, consistent with epithelial injury, whereas FM patients had milder gastrointestinal symptoms and less pronounced biomarker alterations. Although overall PC scores did not significantly differ across groups, serum zonulin levels showed a strong inverse correlation with GPCS. When stratified by GPCS (cut-off ≥6), patients in the high PC group exhibited significantly lower (p = 0.01) serum zonulin concentrations (48.23 ± 12.44 ng/mL) compared to those in the low PC group (57.63 ± 9.69 ng/mL). Multiple regression analysis confirmed GPCS as an independent predictor of serum zonulin (β = −9.67, p = 0.01), while BMI was not a significant contributor (p = 0.79). Furthermore, urinary indole levels correlated positively with both lactulose excretion and the Lac/Man ratio, supporting the existence of a dysbiosis-permeability feedback loop in these disorders.

**Conclusion:**

Intestinal barrier dysfunction in FM and IBS displays phenotype-specific patterns and is significantly modulated by PC. These findings support the integration of PC assessment into clinical phenotyping and highlight potential targets for personalized management of chronic overlapping pain syndromes.

## 1 Introduction

Fibromyalgia (FM) and irritable bowel syndrome (IBS) are two common chronic functional disorders that often occur together, with epidemiological studies showing comorbidity rates between 30% and 70% (Drossman, 2016; Lichtenstein et al., 2018; Erdrich et al., 2020b; Masood et al., 2023). Although these conditions share key clinical features such as chronic pain, fatigue, and gastrointestinal (GI) issues, their underlying mechanisms are not fully understood. While central sensitization and gut-brain axis dysregulation have been well studied (Labanski et al., 2020), recent evidence indicates that intestinal barrier dysfunction might play a crucial role in linking GI and musculoskeletal symptoms in these overlapping disorders (Erdrich et al., 2020a). The integrity of the intestinal barrier, maintained by the coordinated function of tight junction (TJ) proteins, the mucus layer, and mucosal immune surveillance (Okumura and Takeda, 2025), seems compromised in both FM and IBS. Elevated serum zonulin levels, a marker of increased intestinal permeability, have been consistently observed in IBS patients (Linsalata et al., 2018; Seethaler et al., 2021) and, to a lesser extent, in FM populations (Erdrich et al., 2020a). This barrier dysfunction allows luminal components, such as lipopolysaccharides (LPS), to translocate, activating Toll-like receptor 4 -dependent inflammatory cascades that may increase systemic pain and contribute to symptom persistence (Ohira et al., 2017). Multiple factors contribute to intestinal barrier disruption in these conditions. Psychological stress, a well-known exacerbating factor in IBS, has been shown to increase intestinal permeability by modulating TJ proteins via cortisol (Martel et al., 2022). At the same time, intestinal dysbiosis, characterized by a decreased abundance of short-chain fatty acid-producing bacteria, reduces butyrate availability (Canani et al., 2011). This metabolic change impairs histone deacetylase (HDAC) inhibition, leading to upregulation of myosin light chain kinase and phosphorylation of TJ proteins (Peng et al., 2009).

Physical deconditioning is another significant factor, particularly in FM patients, who often exhibit reduced aerobic capacity, decreased muscle strength, and unfavorable body composition compared to healthy controls (Johannesson et al., 2011; Ursini et al., 2011; Góes et al., 2012). This reduced physical capacity (PC) results from pain- and fatigue-driven activity avoidance (Góes et al., 2012), creating a vicious cycle that may further harm gut barrier function by decreasing splanchnic perfusion and altering microbial ecology (Aya et al., 2021; Majnarić et al., 2021). The Global Physical Capacity Score (GPCS), a combined index based on validated performance tests, has become a sensitive tool to measure these functional impairments (Bianco et al., 2023). Notably, higher GPCS scores are associated with better clinical outcomes in IBS (Bianco et al., 2023), potentially indicating improved stress resilience and better control of inflammation (Johannesson et al., 2011; Riezzo et al., 2023). Despite these advances, significant knowledge gaps remain regarding the interactions between the gut and muscle. The comorbid FM + IBS presentation, although clinically common, remains poorly understood at the mechanistic level. Current research approaches typically focus on PC as a treatment outcome rather than exploring its potential as a modulator of intestinal barrier function. Additionally, existing biomarker studies often fail to differentiate between distinct patterns of barrier disruption, such as epithelial injury, reflected by intestinal fatty acid-binding protein (I-FABP), and paracellular permeability, assessed by zonulin or the lactulose/mannitol (Lac/Man) ratio, across different clinical phenotypes (Grootjans et al., 2016; Sturgeon and Fasano, 2016).

The present study was designed to address these limitations by providing a detailed analysis of intestinal barrier dysfunction in FM, IBS, and their overlap. Using multiple biomarkers, including serum and fecal zonulin, I-FABP, and the Lac/Man ratio, in combination with GPCS assessments, we sought to identify specific patterns of gut barrier disruption associated with different phenotypes and their relationships to physical performance measures. Our approach offers new insights into how objective measures of PC may affect intestinal health in functional disorders.

We hypothesized that: (1) FM + IBS comorbidity would show the most significant TJ disruption (increased zonulin and Lac/Man ratio), (2) IBS patients would mainly display epithelial injury markers (higher I-FABP levels), and (3) higher GPCS scores would be associated with better barrier integrity across all groups. These findings could help develop personalized treatments targeting dysfunction of the gut-muscle axis in functional GI and pain disorders.

## 2 Methods

### 2.1 Patient recruitment

Adult patients (aged 18–65 years) with FM and/or IBS were recruited from the medical staff of the “Functional Gastrointestinal Disorders” Outpatients Clinic, the “Rheumatology” Outpatient Clinic, and the “Outpatient Pain Management” Unit of the IRCCS ″S. de Bellis” in Castellana Grotte, Italy.

Regarding the inclusion criteria, FM diagnosis was established according to the 2021 clinical practice guidelines of the Italian Society for Rheumatology (SIR) (Ariani et al., 2021). These national guidelines endorse the American College of Rheumatology (ACR) 2010/2011 diagnostic criteria (Wolfe et al., 2011; Wolfe and Häuser, 2011) as a validated, symptom-based framework for both clinical and research use. Accordingly, all enrolled FM patients fulfilled the ACR 2010/2011 criteria as defined by a Widespread Pain Index (WPI) ≥ 7 and Symptom Severity Scale (SSS) score ≥5, or WPI 4–6 and SSS ≥9, with symptoms persisting for at least 3 months and not better explained by another disorder. Besides, they had to present a clinical phenotype consistent with the SIR diagnostic framework. For IBS patients, diagnosis was based on Rome IV criteria, with a total score ≥125 on the IBS-Severity Scoring System (IBS-SSS), indicating a moderate to severe degree of symptoms (Farrukh, 2022).

Exclusion criteria were: severe cardiac, hepatic, neurological, or psychiatric diseases, as well as GI disorders other than IBS, such as inflammatory bowel disease or diverticular disease. Patients receiving ongoing symptom-targeted therapies, including probiotics, specialized diets, educational interventions, or complementary medicine, were excluded. Any FM or IBS management medications were to be discontinued at least 15 days before enrollment.

**FIGURE 1:**
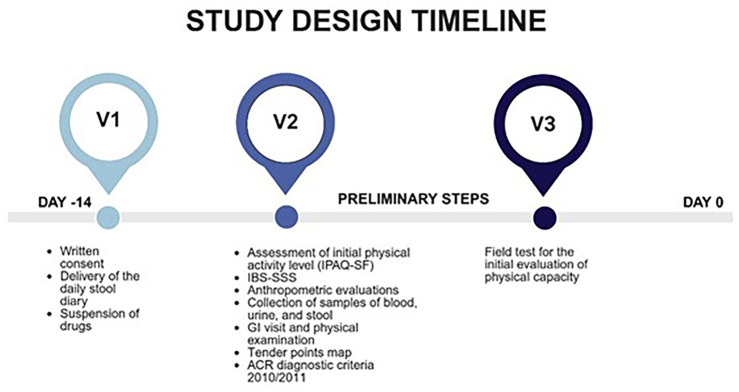
Overview of the study assessment protocol. Timeline and battery of clinical, functional, and laboratory evaluations administered to all participants upon enrollment. IPAQ-SF, International Physical Activity Questionnaire short-form; IBS-SSS, Irritable Bowel Syndrome Severity Scoring System; GI, Gastrointestinal; ACR, American College of Rheumatology.

The present study is part of a broader research project investigating the effects of a physical activity program on symptomatology (GI and psychological) and biochemical parameters in patients with FM and IBS, with or without comorbid conditions. Specifically, the present study focuses on baseline differences in biochemical markers related to intestinal permeability and membrane integrity among patients with FM, IBS, and FM + IBS at study enrollment. Patient recruitment and assessment followed a standardized protocol as outlined in Figure 1.

The research project received approval from both the local Scientific Committee and the Institutional Ethics Committee of the IRCCS Ospedale Oncologico di Bari “Istituto Oncologico Giovanni Paolo II” (Protocol No. 117; Ethics Committee approval granted on March 3, 2023). The trial was conducted following the ethical standards outlined in the 1964 Declaration of Helsinki and its subsequent amendments, and it was prospectively registered at http://www.clinicaltrials.gov under the identifier NCT06166563. The final data access occurred on April 30, 2025.

### 2.2 Assessment of fibromyalgia and gastrointestinal symptoms

The severity of FM symptoms was evaluated using validated questionnaires: a) Fibromyalgia Impact Questionnaire, Revised Version (FIQ-R) (Sarzi-Puttini et al., 2003); b) Visual analog scale pain score (Crawford et al., 2011); c) Fatigue severity scale (Learmonth et al., 2013); d) Digital tender point examination (Cott et al., 1992). Before evaluating GI symptoms, a detailed clinical history was recorded.

The IBS-SSS was administered to assess the GI-symptom profile. The severity of IBS was determined using the generally accepted IBS-SSS cut-off scores: >75–175 for mild IBS, 175–300 for moderate IBS, and >300 for severe IBS (Francis et al., 1997). Additionally, stool characteristics were evaluated using the Bristol Stool Scale (Riegler and Esposito, 2001).

### 2.3 Sugar absorption test for small intestinal permeability

All study participants underwent evaluation of small intestinal permeability using an *in vivo* permeability test, which involved a sugar absorption test following an overnight fast. Before test administration, baseline urine samples were collected in the laboratory to screen for endogenous presence of the target sugars. Subsequently, subjects ingested a test solution containing 10 g of lactulose, 5 g of mannitol, and 40 g of sucrose dissolved in 100 mL of water. The inclusion of sucrose in the test solution was based on established protocols from our previous research (Linsalata et al., 2018) and is intended to provide an additional measure of gastric and proximal small intestinal permeability, as sucrose is not absorbed in healthy individuals and its urinary excretion can indicate upper GI barrier dysfunction. The dose of 40 g was selected to ensure detectable levels while minimizing potential osmotic effects, consistent with prior methodological studies (Linsalata et al., 2018).

Urine was collected over 5 h post-ingestion. Total urine volume for each participant was measured and documented. From each sample, a 2 mL aliquot was taken, centrifuged, and stored at −80 °C until further analysis.

Quantification of the three sugar markers (lactulose, mannitol, and sucrose) in urine was performed by high-performance liquid chromatography using an analytical method previously established by our research team (Linsalata et al., 2018). The percentage excretion of each ingested sugar (%Lac, %Man, and %Suc) was calculated. Additionally, the Lac/Man ratio was determined for each sample. A ratio exceeding 0.03 was considered indicative of altered intestinal permeability (Linsalata et al., 2018).

### 2.4 Biomarkers of intestinal barrier function, integrity, and dysbiosis

Blood and stool samples collected from participants were promptly frozen and stored at −80 °C within 12 h of collection. Zonulin levels in both serum and fecal samples were quantified using ELISA kits provided by Immunodiagnostik AG (Bensheim, Germany), following the manufacturer’s instructions. Serum zonulin concentrations below 48 ng/mL and fecal zonulin levels under 107 ng/mL were classified as within the normal reference range (Russo et al., 2018).

Serum concentrations of I-FABP were measured using commercially available ELISA kits from Thermo Fisher Scientific (Waltham, MA, USA).

Urinary indole levels were quantified as a potential indicator of altered microbial metabolism, using a colorimetric assay (Indican Assay Kit, ABNOVA Corporation, Taipei, Taiwan). Concentrations exceeding 20 mg/L were deemed to indicate increased bacterial activity (Linsalata et al., 2023).

### 2.5 Physical activity and physical capacity evaluation

Physical activity levels were evaluated using the short-form International Physical Activity Questionnaire (IPAQ-SF) (Lee et al., 2011), which categorizes activity into low, moderate, or high based on self-reported data.

Field-based fitness assessments were conducted to evaluate various components of PC. The 2-kilometer walking test (Laukkanen et al., 1992) was used to assess cardiorespiratory capacity, serving as a reliable indicator of cardiovascular health, overall fitness, and metabolic efficiency. The Handgrip Test was used to measure strength. A dynamometer was used to evaluate the maximum isometric strength of the forearm muscles (Pearn and Bullock, 1979). Flexibility was assessed using the sit-and-reach test (Hoeger and Hopkins, 1992), which measures the ability to reach forward while seated and reflects flexibility of the lower back and hamstrings.

Specific precautions were taken during field testing with participants diagnosed with FM, as recommended by the American College of Sports Medicine (Han et al., 2024). Before testing, symptom assessments evaluated pain severity, fatigue levels, and exercise tolerance. Participants were verbally encouraged to perform to the best of their ability while ensuring safety throughout the procedures. Adequate rest periods were incorporated between tests to prevent symptom exacerbation. Continuous monitoring of pain and fatigue was maintained during all assessments. In addition, participants were instructed on how to distinguish post-exercise muscle soreness from fatigue and pain related to FM to avoid misinterpreting symptoms.

Several standardization procedures were established to ensure the reliability and reproducibility of test results. All assessments were conducted under consistent conditions, including location, time of day, personnel, and equipment. Clear instructions and demonstrations were provided before each test, and participants were encouraged to wear comfortable clothing and suitable footwear.

### 2.6 Global Physical Capacity Score

PC was evaluated using motor tests of varying difficulty, previously validated in adult populations, to estimate cardiorespiratory fitness, muscular strength, and flexibility, as detailed in the preceding section. PC scores were determined from each test’s results, using age- and sex-specific normative reference charts. Each physical test was scored 1 to 5, and the scores from the three tests were combined to obtain an overall GPCS, with a possible total score of 3–15. In the absence of a standardized cut-off, we used the previously proposed value of GPCS ≥6 (Verrelli et al., 2025). Thus, to facilitate clinical interpretation, participants were dichotomized based on this cut-off value: ≥6 (high PC group) and <6 (low PC group). This composite scoring approach offers a more comprehensive PC assessment by integrating multiple functional domains relevant to daily life, rather than interpreting each test in isolation.

### 2.7 Statistical analysis

Continuous variables are reported as means ± standard error of the mean (SEM), unless otherwise specified. Categorical data are represented as numbers and percentages. Group comparisons were carried out using non-parametric tests. Correlations between variables were calculated using the Spearman correlation test.

Given the exploratory nature of this descriptive study on the Italian population and the lack of prior data on the expected magnitude of between-group differences, a formal *a priori* sample size calculation was not feasible. This approach aligns with observational studies that aim to generate hypotheses and inform the design of future trials, thereby enhancing the statistical power of those trials.

A multiple linear regression analysis was conducted on the raw data to identify predictors of mucosal integrity, incorporating clinical and biochemical variables as independent predictors through a stepwise selection method. To include categorical variables in the model, dummy variables were created by assigning binary codes (typically 0 and 1). The proportion of variance explained by the model was quantified using the adjusted R-squared, and its overall significance was tested via an F-statistic. The individual contributions of each predictor were assessed using t-statistics and corresponding p-values to determine whether the regression coefficients differed significantly from zero.

All statistical analyses were performed using SigmaStat version 11.0 (Systat Software, Inc., San Jose, CA, USA) and GraphPad Prism version 5 (GraphPad Software, Inc., La Jolla, CA, USA). A p-value below 0.05 was considered indicative of statistical significance.

## 3 Results

### 3.1 Participant characteristics

The study included 56 participants classified into three groups: FM (n = 14), FM + IBS (n = 23), and IBS (n = 19).

**FIGURE 2:**
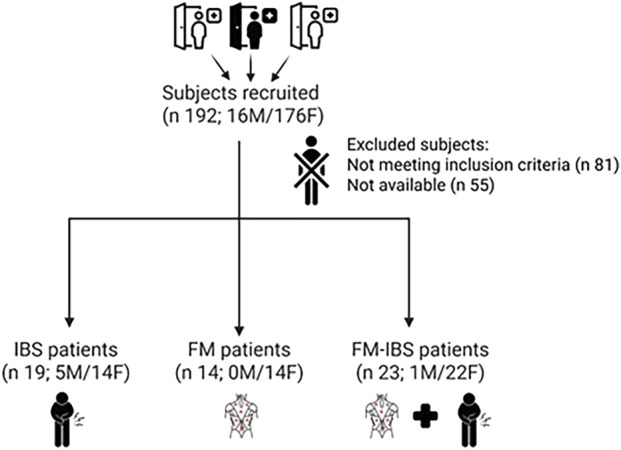
Participant flow through the study. The diagram details the number of individuals screened, eligible, enrolled, and included in the final analysis across the three diagnostic groups: fibromyalgia (FM), irritable bowel syndrome (IBS), and comorbid FM and IBS (FM + IBS).

A flowchart summarizing participant recruitment and group allocation is shown in Figure 2.

Table 1 collects the baseline characteristics of the patients entered in the study. Age was not different among the 3 groups. The mean body mass index (BMI) of participants was within the normal-to-overweight range across all study groups, with no statistically significant intergroup differences detected, consistent with the high prevalence of excess weight reported in populations with chronic conditions, particularly FM. PC evaluation (performed by the GPCS) did not show a statistical difference among groups. The baseline IBS-SSS scores indicated moderate symptom severity in both the IBS (248.3 ± 16.83) and FM + IBS (284.1 ± 16.86) groups, while the FM group reported significantly lower scores (112.6 ± 22.83). Lastly, symptom severity, as measured by the FIQ-R, did not differ between FM and FM + IBS (Table 1). Analysis of physical activity levels using the IPAQ-SF showed a similar distribution among the three groups (FM, FM + IBS, and IBS), with no statistically significant differences.

**TABLE 1:** Baseline characteristics of participants.

NS (not significant)	FM (n.14)	FM + IBS (n. 23)	IBS (n.19)	p
Sex (Male/Female)	0 M/14 F	1 M/22 F	5 M/14 F	
Age (years)	50.4 ± 1.84^a^	44.0 ± 2.03^a^	49.5 ± 2.26^a^	0.07
BMI	27.3 ± 2.19^a^	23.8 ± 0.9	26.9 ± 1.05^a^	0.07
GPCS	6.88 ± 0.66^a^	7.16 ± 0.48^a^	7.60 ± 0.49^a^	0.65
IBS-SSS Total score	112.6 ± 22.83^a^	284.1 ± 16.86^b^	248.3 ± 16.83^b^	<0.0001
FIQ-R-score	64.13 ± 6.46	68.43 ± 6.09	//	0.55
IPAQ Categories°				
<700	5 (35.7%)	8 (34.8%)	7 (36.9%)	
700–2,519	2 (14.3%)	8 (34.8%)	4 (21.0%)	
≥2,520	7 (50.0%)	7 (30.4%)	8 (42.1%)	

BMI, Body Mass Index; GPCS, Global Physical Capacity Score; IBS-SSS, Irritable Bowel Syndrome Severity Scoring System; FIQ-R, Fibromyalgia Impact Questionnaire Revised; IPAQ, International Physical Activity Questionnaire. Continuous data reported as Mean ± SEM. Categorical data are represented as numbers and percentages. At the Kruskal-Wallis with Dunn’s post-test, different letters indicate significant differences. p*: Significance obtained with the Chi-Square test. FIQ-R score analyzed by Mann-Whitney test. °: IPAQ categories expressed in MET (metabolic equivalent of task).

All biochemical data for the patients included in the study remained within normal reference ranges, indicating no clinically relevant alterations between the groups (see Supplementary Table S1).

The assessments of the specific biomarkers of intestinal barrier function (Lac/Man ratio, zonulin, I-FABP) and urinary indole are reported in the following paragraphs.

### 3.2 Intestinal permeability

**FIGURE 3:**
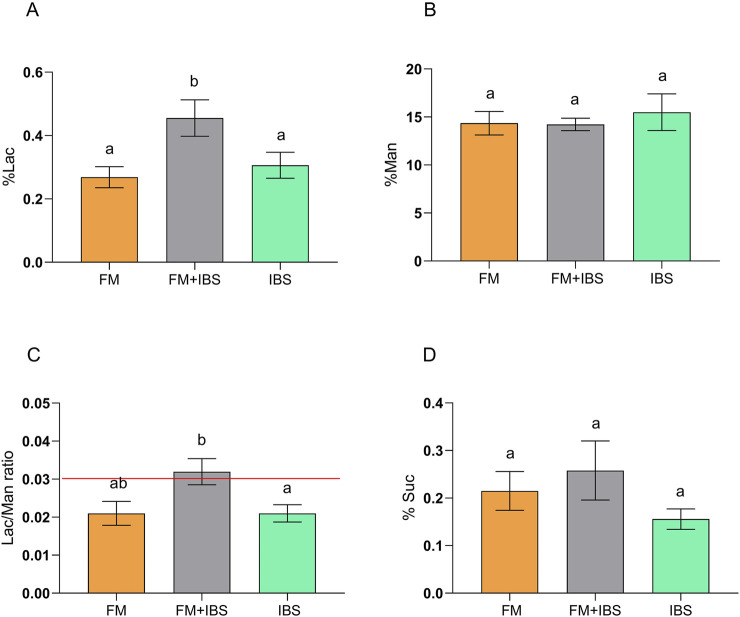
Gastrointestinal permeability assessed by the sugar absorption test in patients with fibromyalgia (FM; n = 14), irritable bowel syndrome (IBS; n = 19), and comorbid FM and IBS (FM + IBS; n = 23). Panel **(A)** Percentage of ingested lactulose recovered in urine (%Lac); Panel **(B)** Percentage of ingested mannitol recovered in urine (%Man); Panel **(C)** Lactulose-to-Mannitol urinary excretion ratio (Lac/Man); Panel **(D)** Percentage of ingested sucrose recovered in urine (%Suc). Data are presented as mean ± standard error of the mean (SEM). Groups were compared using the Kruskal–Wallis test followed by Dunn’s *post hoc* multiple comparisons test; bars labeled with different letters indicate statistically significant differences (p < 0.05). The red line in Panel **(C)** represents the upper limit of the normal reference range for the Lac/Man ratio, as established in healthy control populations.

Intestinal permeability was assessed using the percentages of urinary excretion of the sugar probes (%Lac, %Man, %Suc) and the Lac/Man ratio. Significant differences were observed between groups for %Lac (p = 0.01) and the Lac/Man ratio (p = 0.02). Post-hoc analysis revealed that the FM + IBS group exhibited significantly higher %Lac values (0.46 ± 0.06) compared to both the FM group (0.274 ± 0.03, p = 0.003) and the IBS group (0.31 ± 0.04, p = 0.048). Similarly, the Lac/Man ratio was significantly higher in the FM + IBS group (0.03 ± 0.003) compared to the IBS group (0.02 ± 0.003, p = 0.04) (Figures 3A–D). Finally, no significant differences were observed between the groups in terms of %Suc.

### 3.3 Zonulin levels

**FIGURE 4:**
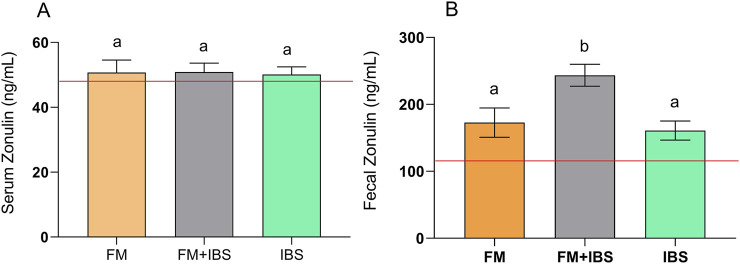
Serum Zonulin (Panel **(A)**) and Fecal Zonulin (Panel **(B)**) concentrations in patients with fibromyalgia (FM; n = 14), irritable bowel syndrome (IBS; n = 19), and comorbid FM and IBS (FM + IBS; n = 23). Data are presented as mean ± SEM. Groups were compared using the Kruskal–Wallis test followed by Dunn’s *post hoc* multiple comparisons test; bars labeled with different letters indicate statistically significant differences (p < 0.05). The red line represents the upper limit of the normal reference ranges for the serum and fecal zonulin levels, as established in healthy control populations.

Both serum and fecal zonulin levels were analyzed as markers of intestinal barrier integrity (Figure 4). No differences were found between the groups concerning serum zonulin levels (Figure 4A). In contrast, significant differences in fecal zonulin levels were observed between groups (p = 0.001). The FM + IBS group exhibited the highest fecal zonulin levels (243.5 ± 16.33 ng/mL), showing a 35.9% increase compared to the FM group (172.8 ± 21.87 ng/mL, p = 0.04) and a 46.0% increase compared to the IBS group (160.80 ± 14.2 ng/mL, p = 0.01) (Figure 4B).

### 3.4 Enterocyte damage and gut dysbiosis

**FIGURE 5:**
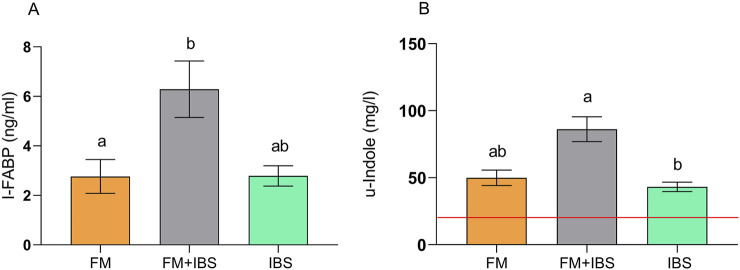
Intestinal fatty acid-binding protein (I-FABP; Panel **(A)**) and urinary indole (u-indole; Panel **(B)**) concentrations in patients with fibromyalgia (FM; n = 14), irritable bowel syndrome (IBS; n = 19), and comorbid FM and IBS (FM + IBS; n = 23). Data are presented as mean ± SEM. Groups were compared using the Kruskal–Wallis test followed by Dunn’s *post hoc* multiple comparisons test; bars labeled with different letters indicate statistically significant differences (p < 0.05). The red line in Panel **(B)** represents the upper limit of the normal reference range for the urinary indole, as established in healthy control populations.

Figure 5A, reports the serum levels of I-FABP, a marker of enterocyte damage. I-FABP levels were significantly elevated in the FM + IBS group (6.29 ± 1.14 ng/mL), showing a 127.5% increase compared to the FM group (2.76 ± 0.68 ng/mL, p = 0.049). No significant difference was observed between the FM and IBS groups (p > 0.05).

Urinary indole levels, an indicator of gut dysbiosis, were significantly higher in the FM + IBS group (86.19 ± 9.22 μg/mL), with a not significant 58.9% increase compared to the FM group (49.96 ± 5.74 μg/mL, p = 0.03) and a significant 99.6% increase compared to the IBS group (43.18 ± 3.52 μg/mL, p < 0.001) (Figure 5B). Lastly, the examination of barrier integrity and dysbiosis parameters revealed a significant positive correlation between the Lac/Man ratio and urinary indole (rho = 0.36, p = 0.01).

### 3.5 Relationship among zonulin, global physical capacity score, body mass index, lactulose/mannitol ratio, and indole

The correlation study on the raw data revealed that BMI was positively correlated with CRP (rho = 0.55, p < 0.001) and inversely correlated with GPCS (rho = −0.37, p = 0.01). Intestinal permeability, expressed as the Lac/Man ratio, correlated with urinary indole concentration, a marker of intestinal dysbiosis (rho = 0.32, p = 0.02). In particular, %Lac, but not %Man, correlated with urinary indole (rho = 0.35, p = 0.01), confirming that dysbiosis alters the paracellular pathway and compromises TJ.

Regression analysis of the raw data revealed that serum zonulin levels could be linearly predicted from both clinical and biochemical variables. Specifically, the GPCS group showed a positive effect on serum zonulin levels, confirming the relationship between PC and mucosal barrier integrity. BMI did not significantly contribute to the equation’s ability to predict serum zonulin (F = 3.29; df = 2; p = 0.045; adjusted R^2^ = 0.08).

In the multivariable regression model, the association between each predictor and the outcome was assessed. BMI showed no statistically significant association with the outcome (β = −0.08, SE = 0.29, p = 0.79; 95% CI: −0.64 to 0.49). In contrast, membership in the GPCS group was significantly associated with a lower outcome score (β = −9.67, SE = 3.82, p = 0.02; 95% CI: −17.25 to −2.28).

Stratifying for GPCS, serum zonulin levels in the whole group were significantly lower in the high PC group than in the low PC group (48.231 ± 1.920 vs. 57.63 ± 2.59, p = 0.01), with an increase of 15.41% in serum zonulin concentration. No significant differences were found among other intestinal permeability markers (fecal zonulin, I-FABP, %Lac, and Lac/Man ratio), urinary indole levels or symptom profile in the other groups (p > 0.05). Data analyzed by Mann-Whitney test. These results demonstrate the importance of assessing multiple mucosal barrier integrity indices to evaluate GPCS in patients.

## 4 Discussion

Our findings establish intestinal barrier dysfunction as a key feature of FM, exacerbated in FM + IBS comorbidity. Notably, serum zonulin levels inversely correlated with PC across all groups (FM, FM + IBS, IBS), supporting the protective role of PC in gut health.

Comparative analysis revealed distinct phenotypic patterns of barrier disruption. FM + IBS patients demonstrated the most severe paracellular permeability, as reflected in elevated fecal zonulin and Lac/Man ratios. No significant differences were observed between the groups in %Suc, suggesting preserved gastric and proximal small intestinal barrier integrity. In contrast, IBS patients exhibited predominantly epithelial injury, as evidenced by increased I-FABP levels.

Additionally, patients with FM reported mild GI symptoms, which were significantly lower than those in the IBS and FM + IBS groups, both of which showed moderate IBS-SSS total scores.

These phenotypic differences imply distinct pathogenic mechanisms: FM-related barrier dysfunction likely reflects dysregulation of TJ proteins (stress-mediated and inflammatory pathways). At the same time, IBS appears driven by epithelial injury (mast cell-mediated hypersensitivity) (Hasler et al., 2022). Such mechanistic divergence underscores the necessity for phenotype-stratified therapies targeting specific barrier defects.

The microbial metabolite indole emerged as a key microbial metabolite, with FM + IBS patients showing significantly higher levels than those with FM. It is important to note that urinary indole levels reflect a complex interplay between gut microbial tryptophan metabolism and intestinal barrier integrity. Furthermore, their concentration is strongly influenced by dietary tryptophan intake (Kim et al., 2025). In the absence of detailed dietary records or metagenomic profiling, elevated indole excretion should be interpreted cautiously as a standalone marker of dysbiosis. Future studies should integrate dietary monitoring and microbiome analysis to better contextualize these metabolic signals.

Notably, the positive correlation between urinary indole and the Lac/Man ratio supports the concept of a dysbiosis-permeability loop, in which microbial imbalances exacerbate barrier dysfunction, thereby fueling systemic inflammation and nociceptive sensitization (Singh et al., 2021). This vicious cycle could be critical in FM + IBS patients, in whom central sensitization and visceral hypersensitivity may intensify the effects of dysbiosis and barrier disruption (Ho et al., 2025). In this framework, the dual role of indole, as both a marker of dysbiosis and a modulator of intestinal permeability, makes this finding particularly compelling (Roager and Licht, 2018).

Our findings identify distinct therapeutic opportunities that align with specific phenotypic mechanisms. In FM + IBS, targeted barrier restoration, potentially through glutamine supplementation to modulate zonulin-dependent permeability, may be prioritized, consistent with its efficacy in other permeability disorders (Arribas-López et al., 2021). For IBS cases, mast cell stabilizers such as ketotifen are rational candidates given their demonstrated epithelial-protective effects in similar cohorts (Fortea et al., 2021). Conversely, FM patients may benefit most from combined stress-axis modulation and TJ stabilization, addressing cortisol-mediated barrier dysregulation (Ohira et al., 2017). This mechanistic stratification underscores the potential for precision management of gut-muscle axis dysfunction. In this perspective, our data may also serve as a preliminary foundation for the future development of a translational decision-making algorithm to guide clinicians in a phenotype-oriented management of FM and IBS. At the current stage, however, these findings should be regarded as exploratory, and further validation in larger cohorts, incorporating additional clinical and molecular parameters, will be required before this approach can be translated into routine clinical practice.

Furthermore, our study reveals a novel association between physical deconditioning (lower GPCS) and elevated serum zonulin, suggesting that reduced PC contributes to intestinal barrier dysfunction. Notably, this association was specific to serum zonulin, which may reflect systemic barrier disruption more sensitively than fecal zonulin, I-FABP, or %Lac. Unlike BMI, a crude metabolic surrogate, GPCS provides a functional assessment of physical status and correlates more strongly with zonulin-mediated barrier impairment. Mechanistically, PC may influence barrier integrity through splanchnic hypoperfusion, oxidative stress, and Hypotalamic-Pituitary-Adrenal (HPA) axis dysregulation (de Oliveira and Burini, 2009; Bilski et al., 2018), which collectively promote cortisol-driven zonulin release, downregulation of TJ proteins (e.g., occludin, claudin), and microbial dysbiosis (Panwar et al., 2021; Riezzo et al., 2023).

The observed trends support the hypothesis that greater PC may promote improved gut health. In this context, it has been demonstrated that enhanced cardiovascular fitness may improve splanchnic perfusion, thereby reducing intestinal hypoxia and oxidative stress, which are known to compromise the TJ integrity (Keirns et al., 2020). Additionally, physical exercise promotes microbial diversity, particularly enriching butyrate-producing species such as Faecalibacterium prausnitzii, which strengthens the epithelial barrier through HDAC Inhibition (Zhang et al., 2021) and upregulation of occludin (O'Riordan et al., 2022). Finally, exercise modulates the HPA axis, potentially reducing cortisol-mediated zonulin release (Clark and Mach, 2016).

Physical exercise can be viewed as a pathogenically targeted intervention, akin to pharmacological agents, with dose-dependent and phenotype-specific effects. Its ability to modulate intestinal, inflammatory, and functional biomarkers positions it as both a preventive strategy and a primary therapeutic approach, particularly when tailored to individual patient characteristics. This aligns with the principles of precision medicine, which aim to tailor interventions based on individual biological and functional profiles. Integrating non-invasive biomarkers (e.g., zonulin, I-FABP) with physical performance indices, such as the GPCS, could facilitate a shift from symptom-based management to mechanism-driven stratification and care. Phenotypic stratification guided by such biomarkers may enable a precision medicine approach. For example, a patient with FM + IBS, high zonulin, and low GPCS might receive a combined regimen of graded exercise, probiotics targeting zonulin production (e.g., *Lactobacillus* rhamnosus GG, which has shown efficacy in modulating gut permeability (Chen et al., 2019), and gut-directed nutrients. In contrast, an IBS patient with elevated I-FABP may initially benefit from mast cell modulation and epithelial repair agents, such as zinc carnosine (Mahmood et al., 2007). This paradigm shift from symptom management to mechanistic targeting aligns with emerging frameworks for complex chronic disorders (Clauw, 2014).

While we advocate for the integration of PC assessment and exercise-based interventions, it is imperative to acknowledge that fatigue is a core and often debilitating symptom of FM. Any exercise program must be carefully graded and individualized, starting at a very low intensity and gradually increasing, to avoid exacerbating fatigue and pain. Patient education on pacing and energy conservation strategies is equally crucial to ensure adherence and prevent symptom flare-ups.

Although our study provides critical new insights into the gut-muscle axis in FM, several limitations must be acknowledged. Most notably, the cross-sectional design precludes causal inference regarding the relationships among PC, intestinal barrier function, and clinical symptom severity. As such, while our results reveal significant associations, they do not clarify whether reduced PC promotes barrier dysfunction or if mucosal alterations contribute to fatigue and deconditioning. Future longitudinal or interventional studies are needed to establish causality and determine whether targeted improvements in physical fitness lead to measurable benefits for gut barrier function.

Second, the absence of a healthy control group limits the generalizability of our findings. However, the sample included individuals from diverse geographic and socioeconomic backgrounds in Southern Italy, thereby enhancing external validity despite being recruited from a single center. Stratification by clinical phenotype, along with comparisons to published reference values for zonulin and I-FABP, allowed us to identify meaningful intergroup differences. Future studies should include healthy controls to establish baseline levels for intestinal barrier biomarkers and strengthen comparative analyses. A significant limitation of this study is the lack of formal classification of IBS subtypes (e.g., diarrhea-predominant, constipation-predominant). As different IBS subtypes may exhibit distinct patterns of barrier dysfunction and dysbiosis (Linsalata et al., 2018), this represents a crucial area for future investigation to refine phenotype-specific therapeutic approaches.

The acknowledged limitation of uncontrolled dietary intake must temper the interpretation of fecal zonulin levels. As dietary components, particularly gluten, have been shown to modulate fecal zonulin (Fasano, 2020), the observed elevations in the FM + IBS group may reflect dietary habits rather than, or in addition to, intrinsic barrier dysfunction. This highlights the importance of dietary standardization in future research utilizing this biomarker.

Third, dietary intake, a known influence on gut permeability and microbiota composition, was not systematically assessed. This omission may confound the interpretation of indole and zonulin levels. Future studies should include validated dietary questionnaires to address this limitation. Additionally, while anthropometric assessment (BMI) confirmed the link between FM and overweight/obesity (Ursini et al., 2011), our regression model demonstrated that BMI does not affect intestinal permeability in these patients.

Another important limitation is the potential confounding effect of sex. Given the known influence of sex hormones on intestinal epithelial permeability (Larauche et al., 2025) and the higher female prevalence of FM (Daher et al., 2024) and IBS (Kim and Kim, 2018), the observed differences in biomarkers might be partially attributable to sex distribution rather than disease phenotype alone. Future studies should aim for balanced sex representation or perform sex-stratified analyses.

Finally, some limitations affect the interpretation of zonulin: current ELISA assays lack specificity due to cross-reactivity with properdin and may detect non-haptoglobin-2 isoforms (Scheffler et al., 2018). To mitigate this, we contextualized zonulin data with I-FABP and sugar absorption tests. Similarly, while urinary indole shows promise as a non-invasive marker of dysbiosis, its pathophysiological relevance requires validation through targeted metabolomics and microbiome-based analyses.

In conclusion, this study demonstrates that intestinal barrier dysfunction is a shared yet phenotype-specific hallmark of FM and IBS, with the most severe impairment in comorbid cases. Distinct mechanisms (i.e., TJ dysfunction in FM and epithelial injury in IBS) underscore the need for personalized approaches. The observed inverse relationship between PC and barrier dysfunction underscores the potential to integrate exercise-based interventions and biomarker-guided stratification in the management of these complex functional disorders.

## Data Availability

The datasets presented in this study can be found in online repositories. The names of the repository/repositories and accession number(s) can be found below: The data presented in this study are openly available at 10.6084/m9.figshare.29656031.
