# Supplementary material for: Physical capacity modulates intestinal barrier dysfunction in functional disorders: phenotype-specific patterns in fibromyalgia and irritable bowel syndrome
Source: Front Physiol. 2025 Oct 29;16:1695163. doi: 10.3389/fphys.2025.1695163 (PMC12604992; doi:10.3389/fphys.2025.1695163)
Supplement: Supplementary file 1 [file Table1.docx]

**Table S1.** Biochemical characteristics of the participants

|  | **FM (n.14)** | **FM-IBS (n.23)** | **IBS (n.19)** | p |
| --- | --- | --- | --- | --- |
| TSH (μU/mL) | 2.34 ± 0.29^a^ | 2.06 ± 0.17^a^ | 2.04 ± 0.24^a^ | 0.63 |
| fT3 (pg/mL) | 3.33 ± 0.10^a^ | 2.97 ± 0.12^a^ | 3.27 ± 0.09^a^ | 0.06 |
| fT4 (pg/mL) | 1.19 ± 0.04^a^ | 1.24 ± 0.05^a^ | 1.24 ± 0.03^a^ | 0.57 |
| Glucose (mg/dL) | 84.2 ± 3.14^ab^ | 78.4 ± 1.79^a^ | 86.0 ± 1.66^b^ | 0.02 |
| Insulin (μU/mL) | 8.38 ± 2.12^a^ | 5.97 ± 0.60^a^ | 9.92 ± 1.95^a^ | 0.15 |
| 25-OH-Vit. D (ng/mL) | 35.6 ± 4.55^a^ | 28.1 ± 2.37^a^ | 29.6 ± 2.44^a^ | 0.21 |
| Vit. B12 (ng/mL) | 536.0 ± 48.4^a^ | 447.3 ± 33.1^a^ | 501.3 ± 53.2^a^ | 0.37 |
| γGT (U/L) | 25.1 ± 6.61^a^ | 19.1 ± 3.62^a^ | 19.9 ± 2.51^a^ | 0.59 |
| ALT (U/L) | 21.5 ± 2.64^a^ | 14.2 ± 1.31^b^ | 18.2 ± 1.85^ab^ | 0.03 |
| AST (U/L) | 21.7 ± 1.98^a^ | 16.6 ± 1.08^b^ | 19.5 ± 1.07^ab^ | 0.03 |
| CRP (mg/dL) | 0.3 ± 0.1^ab^ | 0.19 ± 0.06^a^ | 0.29 ± 0.05^b^ | 0.03 |
| Total Cholesterol (mg/dL) | 193 ± 10.6^a^ | 179 ± 7.53^a^ | 203 ± 6.90^a^ | 0.09 |
| HDL Cholesterol (mg/dL) | 70.6 ± 5.93^a^ | 60.3 ± 3.59^a^ | 55.9 ± 2.52^a^ | 0.06 |
| Triglycerides (mg/dL) | 84.2 ± 9.67^a^ | 80.2 ± 9.16^a^ | 104 ± 10.2^a^ | 0.18 |

TSH: thyroid-stimulating hormone; fT3: free triiodothyronine; fT4: free thyroxine; γGT: gamma glutamyl transferase; ALT: alanine aminotransferase; AST: aspartate aminotransferase; CRP: C-reactive protein; HDL Cholesterol: High-Density Lipoprotein Cholesterol**.** Data are expressed as Means ± SEM. The Kruskal-Wallis test with Dunn's multiple-comparison tests was used to assess differences among groups.
